# Changes in Serving Size, Calories, and Sodium Content in Processed Foods From 2009 to 2015

**DOI:** 10.5888/pcd15.170265

**Published:** 2018-03-15

**Authors:** Jenifer E. Clapp, Sarah A. Niederman, Elizabeth Leonard, Christine J. Curtis

**Affiliations:** 1 New York City Department of Health and Mental Hygiene, Long Island City, New York

## Abstract

**Introduction:**

Approximately 60% of the American diet comes from processed foods, which makes improving their nutritional quality important for Americans’ health. The objective of this study was to measure changes in serving sizes, calories, and sodium in top-selling processed foods that were on the market in 2009 and 2015.

**Methods:**

We analyzed products in the top 80% of sales in the 54 processed food categories with consistent serving sizes and sales metrics that were on the market in both 2009 and 2015. Mean serving size, calories (per serving and density), sodium (per serving and density), and sales were calculated for 2,979 branded processed food products. For each stratification of calorie density and sodium density (decreased, increased, or did not change), we calculated the mean serving size, calorie density, sodium density, and sales for each year.

**Results:**

From 2009 to 2015, we found decreases in serving size (−2.3%, *P* < .001), calories per serving (−2.0%, *P* < .001), calorie density (−1.1%, *P* < .001), sodium per serving (−7.6%, *P* < .001), and sodium density (−6.0%, *P* < .001). A decrease in calorie density did not correspond to an increase in sodium density or vice versa. A decline in sales was observed regardless of whether calorie density or sodium density decreased, increased, or did not change.

**Conclusion:**

Reductions in calorie and sodium density occurred in tandem, suggesting that manufacturers reformulated for more than one health goal at the same time. Instead of unintended negative consequences of encouraging companies to reformulate for one nutrient, an overall net nutritional benefit occurred.

## Introduction

Diet influences a person’s risk for overweight, obesity, type 2 diabetes, and hypertension, which are risk factors for cardiovascular disease, the leading cause of death in the United States (1). Over-consumption of processed foods that contain empty calories from added sugars or saturated fats and contain excess sodium has detrimental health effects (1, 2). Approximately 60% of the calorie and sodium intake of adult Americans is from processed foods bought from stores (3, 4). Despite the focus of public health leaders on changes in the food supply as a strategy to influence the nutritional intake of Americans, existing research that monitors the nutritional quality of food is limited and has typically focused on changes in a single nutrient or on multiple nutrients at a single point in time. The objective of our study was to measure changes in serving sizes, calories, and sodium among top-selling processed foods that were on the market in both 2009 and 2015. A secondary objective was to assess whether a decrease in calorie density corresponded to an increase in sodium density and vice versa, and whether those changes were associated with changes in sales.

## Methods

### Sample selection

We derived the sample used for our analysis from the National Salt Reduction Initiative (NSRI) Packaged Food Database. NSRI is the first organized public health effort in the United States to engage industry in reducing sodium in the food supply. A coalition of more than 100 local public health agencies and national health organizations led by the New York City Department of Health and Mental Hygiene encouraged food companies to meet NSRI sodium reduction targets (5). The NSRI database was designed to assess sodium levels and other information listed on nutrition labels of US processed foods over time. Monitoring began in January 2009 and occurred at the deadlines (January 2012 and December 2014) set for companies to voluntarily meet NSRI sodium reduction targets.

The NSRI database profiles nutritional content of products in 62 processed food categories that contribute to sodium intake, such as breads and rolls, cold cuts, and cheese, at 3 points in time. Annual equivalized sales data were purchased from Nielsen, Inc, for these food categories for 2008, 2011, and 2014. The equivalized sales metric converts the various sizes in which a product is sold into one standard unit to uniformly quantify the product’s sales volume. We purchased nutrition data from Guiding Stars Licensing Company and collected it from manufacturer websites and supermarkets in 2009, 2012, and 2015 for products in the top 80% of a food category’s sales in each year.

Not all products in the NSRI database are included in our analysis. Nielsen does not provide product identifiers for private-label products, also known as store brands; therefore, nutrition information could be obtained only for branded products. The number of private-label products varied by category. In 2015 they accounted for 28% of products in the NSRI database. In addition, only products in 54 of 62 food categories reported serving sizes in grams and sales data in equivalized sales, thus allowing for comparisons. Therefore products in 8 categories were not included in our analysis: 6 categories (Asian-style condiments, dry soup mixes, vegetable juice, dry seasoning mixes, seasoned pasta and stuffing, and seasoned grain mixes) reported serving sizes in a volume metric (eg, tablespoons, mL); one category (uncooked whole muscle meat) did not have unit sales data available; and one category (tortillas and wraps) had sales information in a different metric (sales dollars). The remaining 54 food categories span the following broader food categories or metacategories: bakery products; cereal and other grain products; meats; dairy products and substitutes; fats and oils; sauces, dips, gravies, and condiments; snacks; soups; potatoes; mixed dishes; vegetables; legumes; canned fish; and nut butters. A detailed description of the NSRI processed food categories is published elsewhere (5).

To focus on reformulation of the same products over time, branded products on the market in both 2009 and 2015 were matched by universal product codes. The universal product code was used to determine that a product was the same in both years. Products were included in this analysis only when nutrition information was available in both years. A total of 2,979 branded products were on the market in 2009 and 2015 and had gram weight, sodium, and calorie information. This sample represents 79% of all matched branded products (n = 3,794), 40% of branded products in 2009 (n = 7,509) and 36% of branded products in 2015 (8,351).

### Statistical analysis

Mean serving size (grams), calories per serving, sodium (milligrams) per serving, and sales (equivalized units) were calculated for 2009 and for 2015 for the entire sample and by metacategory. Mean calorie and sodium content was calculated per 100 g of food (density) in 2009 and 2015 to standardize the serving size to focus on reformulation. Percentage change was calculated for the entire sample and by metacategory. Next, we determined the number of products in which 4 measures (serving size, calorie density, sodium density, and sales) decreased, increased, or did not change. No change was defined as a percentage change of plus or minus 1% from 2009 to 2015. For each stratification of calorie density and sodium density, the mean serving size, calorie density, sodium density, and sales were calculated in each year along with percentage change. For these analyses, paired *t* tests were used to test whether the difference in means between years was significant.

To further evaluate whether product reformulation was associated with changes in sales, independent sample *t *tests assessed whether the mean difference in sales from 2009 to 2015 was significant between 1) products that decreased versus increased in calorie density from 2009 to 2015 and 2) products that decreased versus increased in sodium density from 2009 to 2015. All *t* tests used a 2-tailed α of .05, and all analyses were completed with SAS version 9.4 (SAS Institute, Inc).

## Results

Mean (standard deviation [SD]) serving size declined significantly by 2.3% (*P* < .001) from 93.1 g (85.1) in 2009 to 90.9 g (82.7) in 2015 (Table 1). From 2009 to 2015 serving size decreased among 14% of products (n = 430), increased among 10% of products (n = 312), and did not change among 75% of products (n = 2,237).

**Table 1:** Comparison of Serving Size, Calories per Serving, and Sodium per Serving Among Top-Selling Processed Food Products in the United States, 2009 and 2015

Food Product	No. (%) of Units	2009, Mean (SD)	2015, Mean (SD)	% Change	*P* Value^a^
**Serving size, g**					
Overall	2,979 (100)	93.1 (85.1)	90.9 (82.7)	−2.3	<.001
Bakery products	520 (17)	44.1 (19.6)	45.0 (19.4)	2.0	.01
Cereal and other grain products	78 (3)	34.6 (9.8)	34.7 (9.6)	0.5	.68
Meats	396 (13)	50.5 (21.0)	50.3 (21.3)	−0.4	.60
Dairy products and substitutes	226 (8)	44.7 (36.9)	44.2 (36.6)	−1.2	<.001
Fats and oils	212 (7)	27.2 (6.6)	27.1 (6.8)	−0.3	.58
Sauces, dips, gravies, condiments	274 (9)	57.2 (39.4)	57.1 (39.3)	−0.1	.50
Snacks	150 (5)	28.7 (2.1)	28.8 (2.9)	0.2	.73
Soups	154 (5)	239.2 (19.2)	211.7 (72.1)	−11.5	<.001
Potatoes	57 (2)	79.7 (37.5)	80.0 (35.3)	0.4	.71
Mixed dishes	621 (21)	195.7 (93.4)	191.0 (90.1)	−2.4	<.001
Vegetables	155 (5)	117.0 (23.7)	120.3 (40.7)	2.9	.24
Legumes	106 (4)	127.6 (4.1)	127.7 (4.0)	0.0	.55
Canned fish	11 (0)	57.3 (2.8)	57.4 (2.8)	0.2	.17
Nut butters	19 (1)	32.5 (1.2)	32.2 (0.6)	−0.8	.10
**Calories, kcal, per labeled serving size**					
Overall	2,979 (100)	145.7 (109.5)	142.9 (101.9)	−2.0	<.001
Bakery products	520 (17)	138.6 (59.7)	141.2 (60.5)	1.9	.01
Cereal and other grain products	78 (3)	126.4 (34.4)	127.4 (34.2)	0.8	.20
Meats	396 (13)	121.5 (63.6)	120.0 (62.6)	−1.2	.15
Dairy products and substitutes	226 (8)	91.4 (23.9)	89.9 (24.9)	−1.7	.04
Fats and oils	212 (7)	99.4 (40.6)	95.7 (41.1)	−3.7	<.001
Sauces, dips, gravies, condiments	274 (9)	42.3 (33.2)	41.1 (31.2)	−2.8	.01
Snacks	150 (5)	134.7 (22.2)	134.3 (22.3)	−0.3	.37
Soups	154 (5)	94.5 (51.1)	93.6 (49.6)	−1.0	.26
Potatoes	57 (2)	128.9 (41.0)	129.2 (43.2)	0.2	.93
Mixed dishes	621 (21)	298.4 (124.5)	284.9 (107.7)	−4.5	<.001
Vegetables	155 (5)	47.4 (30.2)	48.6 (31.7)	2.5	.16
Legumes	106 (4)	111.4 (25.8)	114.6 (25.0)	2.8	<.001
Canned fish	11 (0)	64.1 (15.9)	61.4 (15.2)	−4.3	.17
Nut butters	19 (1)	186.8 (15.3)	191.6 (7.6)	2.5	.20
**Sodium, mg, per labeled serving size**					
Overall	2,979 (100)	419.2 (284.1)	387.3 (248.5)	−7.6	<.001
Bakery products	520 (17)	205.3 (104.4)	197.2 (103.0)	−4.0	<.001
Cereal and other grain products	78 (3)	186.3 (72.1)	165.9 (60.2)	−11.0	<.001
Meats	396 (13)	482.3 (185.2)	460.3 (173.2)	−4.6	<.001
Dairy products and substitutes	226 (8)	249.1 (113.8)	239.6 (112.3)	−3.8	<.001
Fats and oils	212 (7)	260.6 (117.5)	239.2 (100.5)	−8.2	<.001
Sauces, dips, gravies, condiments	274 (9)	309.0 (160.2)	291.0 (137.4)	−5.8	<.001
Snacks	150 (5)	255.8 (133.9)	235.6 (124.8)	−7.9	<.001
Soups	154 (5)	765.3 (179.7)	688.1 (170.3)	−10.1	<.001
Potatoes	57 (2)	368.5 (157.4)	344.7 (157.8)	−6.5	.02
Mixed dishes	621 (21)	747.9 (299.7)	672.0 (237.0)	−10.2	<.001
Vegetables	155 (5)	298.2 (143.5)	269.4 (142.0)	−9.7	<.001
Legumes	106 (4)	446.5 (109.6)	438.6 (116.4)	−1.8	.046
Canned fish	11 (0)	222.7 (42.2)	189.1 (43.2)	−15.1	.03
Nut butters	19 (1)	141.3 (16.3)	143.4 (14.2)	1.5	.49

a Paired *t* tests determined the difference in mean values between 2009 and 2015. *P* values below .05 are significant.

**Figure 1:**
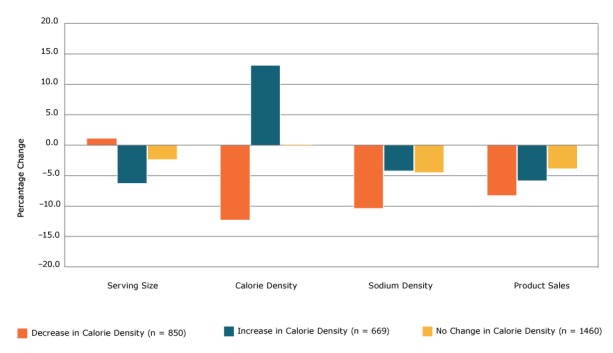
Percentage change in serving size, calorie density, sodium density, and sales among 2,979 processed food products, by level of change in calorie density, 2009 to 2015. Paired *t* tests were used to test whether the difference in means between 2009 and 2015 was significant (2-tailed α of .05). All bars represent significant (P < .05) differences except the leftmost bar within Serving Size (the percentage change in serving size among items that decreased in calorie density). **Table:** 

There was a significant reduction in mean calories in both the per-serving and the density metrics from 2009 to 2015. Mean calories per serving declined significantly (*P* < .001) by 2.0% (mean [SD] kcal, 145.7 [109.5] in 2009 vs mean [SD] kcal, 142.9 [101.9] in 2015) (Table 1), and mean (SD) calorie density declined significantly (*P* < .001) by 1.1% (234.7 [157.4] kcal/100 g in 2009 vs 232.1 [153.8] kcal/100 g in 2015) (Table 2). Calorie density decreased among 29% of products (n = 850), increased among 22% of products (n = 669), and did not change among 49% of products (n = 1,460). Of the 14 metacategories, 2 declined significantly in calorie density and one increased significantly (Table 2). Products that decreased calorie density demonstrated a significant 10.3% (*P* < .001) decline in mean (SD) sodium density, from 614.1 (378.8) mg/100 g in 2009 to 551.1 (347.2) mg/100 g in 2015. Products that increased or did not change calorie density had significant declines in mean (SD) sodium density during the same period (Figure 1) (products that increased calorie density, 4.2% decline [*P* < .001], from 561.3 [345.3] mg/100 g in 2009 to 537.9 [325.1] mg/100 g, in 2015; and products that had no change in calorie density, 4.4% [*P* < .001], from 641.5 [390.8] mg/100 g in 2009 to 613.4 [379.3] mg/100 g in 2015).

**Table 2:** Comparison of Calorie Density and Sodium Density Among Top-Selling Processed Food Products in the United States, 2009 and 2015

Food Product	Calories, kcal/100 g					Sodium, mg/100 g				
	No. (%)	2009, Mean (SD)	2015, Mean (SD)	% Change	*P* Value^a^	No. (%)	2009, Mean (SD)	2015, Mean (SD)	% Change	*P* Value^a^
Overall	2,979 (100)	234.7 (157.4)	232.1 (153.8)	−1.1	<.001	2,979	615.7 (378.8)	578.7 (360.2)	−6.0	<.001
Bakery products	520 (17)	328.7 (104.6)	327.2 (95.4)	−0.5	.42	520 (17)	480.7 (197.9)	447.6 (179.8)	−6.9	<.001
Cereal and other grain products	78 (3)	369.3 (50.4)	369.1 (34.8)	−0.1	.96	78 (3)	566.9 (210.9)	500.8 (175.1)	−11.7	<.001
Meats	396 (13)	266.1 (131.5)	264.2 (130.6)	−0.7	.32	396 (13)	1059.2 (397.7)	1014.8 (385.9)	−4.2	<.001
Dairy products and substitutes	226 (8)	289.5 (117.9)	287.5 (117.7)	−0.7	.18	226 (8)	740.4 (391.9)	717.8 (371.5)	−3.1	<.001
Fats and oils	212 (7)	384.1 (159.2)	371.5 (161.4)	−3.3	<.001	212 (7)	930.1 (306.5)	861.2 (246.5)	−7.4	<.001
Sauces, dips, gravies, condiments	274 (9)	81.4 (66.7)	81.2 (66.4)	−0.3	.77	274 (9)	636.8 (272.8)	613.4 (273.1)	−3.7	<.001
Snacks	150 (5)	469.4 (69.8)	467.2 (66.6)	−0.5	.44	150 (5)	888.5 (466.2)	823.4 (449.8)	−7.3	<.01
Soups	154 (5)	38.8 (20.6)	38.2 (20.4)	−1.6	.13	154 (5)	320.8 (75.2)	286.1 (86.4)	−10.8	<.001
Potatoes	57 (2)	193.6 (99.2)	192.3 (99.0)	−0.7	.66	57 (2)	675.6 (675.6)	624.2 (622.1)	−7.6	.01
Mixed dishes	621 (21)	176.6 (87.4)	171.7 (70.8)	−2.8	.02	621 (21)	436.2 (175.3)	402.9 (153.3)	−7.6	<.001
Vegetables	155 (5)	40.6 (22.8)	41.0 (23.7)	1.0	.63	155 (5)	256.9 (115.8)	228.9 (116.7)	−10.9	<.001
Legumes	106 (4)	87.1 (19.0)	89.5 (18.3)	2.8	<.001	106 (4)	349.5 (84.1)	343.2 (89.2)	−1.8	.04
Canned fish	11 (0)	111.2 (23.1)	106.3 (22.3)	−4.4	.17	11 (0)	388 (65.9)	327.3 (58.2)	−15.6	.03
Nut butters	19 (1)	576.8 (57.8)	595.2 (30.8)	3.2	.15	19 (1)	435.1 (46.6)	445 (40.2)	2.3	.33

a Paired *t* tests determined the difference in mean values between 2009 and 2015. *P* values below .05 are significant.

Change in Calorie Density	No. (%) of Products	2009, Mean (SD)	2015, Mean (SD)	% Change	*P* Value
**Serving size, g**					
Decrease	850 (29)	116.9 (101)	118.1 (102.5)	1.1	.21
Increase	669 (22)	113.3 (90.6)	105.8 (83.3)	−6.6	<.001
No change	1,460 (49)	70.0 (63.4)	68.3 (60.1)	−2.4	<.001
**Calorie density, kcal/100 g**					
Decrease	850 (29)	232.4 (154.3)	203.8 (131.4)	−12.3	<.001
Increase	669 (22)	188.1 (126.6)	213.0 (140.8)	13.2	<.001
No change	1,460 (49)	257.3 (167.0)	257.3 (167.0)	0.0	<.01
**Sodium density, mg/100 g**					
Decrease	850 (29)	614.1 (378.8)	551.1 (347.2)	−10.3	<.001
Increase	669 (22)	561.3 (345.3)	537.9 (325.1)	−4.2	<.001
No change	1,460 (49)	641.5 (390.8)	613.4 (379.3)	−4.4	<.001
**Sales, annual equivalized**					
Decrease	850 (29)	4,307,499.3 (3,609,349)	3,955,056.7 (3,460,379)	−8.2	<.001
Increase	669 (22)	4,478,441.1 (3,498,997)	4,214,948.1 (3,098,732)	−5.9	.02
No change	1,460 (49)	4,412,645.6 (3,596,791)	4,253,249.2 (3,613,180)	−3.6	.02

**Figure 2:**
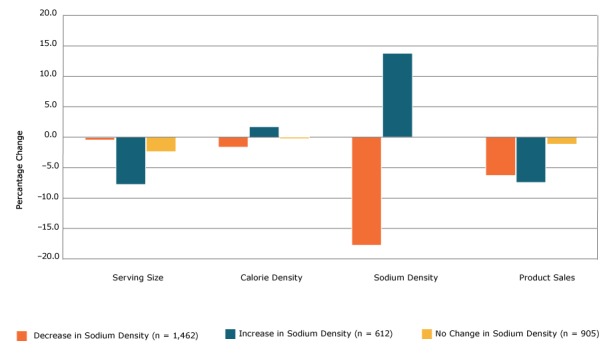
Percentage change in serving size, calorie density, sodium density, and sales among 2,979 processed food products, by level of change in sodium density, 2009 to 2015. Paired *t* tests were used to test whether the difference in means between 2009 and 2015 was significant (2-tailed α of .05). All bars represent significant (*P* < .05) differences except the leftmost bar in Serving Size (the percentage change in serving size among items that decreased in sodium density) and the rightmost bars within Sodium Density and Sales (the percentage change in sodium density among items that did not change in sodium density or sales). **Table:** 

We also saw a significant reduction in mean sodium in both the per-serving (Table 1) and the density metrics (Table 2) from 2009 to 2015. Mean (SD) sodium per serving declined significantly by 7.6% (*P* < .001; 419.2 [284.1] mg in 2009 to 387.3 [248.5] mg in 2015), and mean (SD) sodium density declined significantly by 6.0% (*P* < .001; 615.7 [378.8] mg/100 g in 2009 to 578.7 [360.2] mg/100 g in 2015). Sodium density decreased in 49% of products (n = 1,462), increased in 21% of products (n = 612), and did not change in 30% of products (n = 905). Thirteen of the 14 metacategories had significant declines in sodium per serving and sodium density, and no metacategories had significant increases in sodium per serving or sodium density (Table 1, Table 2). Products that decreased sodium density demonstrated a significant 2.9% (*P* < .001) decline in mean (SD) calorie density (Figure 2), from 224.4 (154.2) kcal/100 g in 2009 to 217.8 (146.2) kcal/100 g in 2015, whereas products that increased sodium density had a significant 2.4% (*P* < .01) increase in mean (SD) calorie density during the same period (216.8 [141.2] kcal/100 g in 2009 vs 222.1 [144.0] kcal/100 g in 2015).

Change in Sodium Density	No. (%) of Units	2009, Mean (SD)	2015, Mean (SD)	% Change	*P* Value
**Serving size, g**					
Decrease	1,462 (49)	105.4 (93.6)	105.0 (93.4)	−0.3	.59
Increase	612 (21)	101.6 (85.3)	94.4 (77.1)	−7.0	<.001
No change	905 (30)	67.5 (61.8)	65.8 (58.4)	−2.5	<.001
**Calorie density, kcal/100 g**					
Decrease	1,462 (49)	224.4 (154.2)	217.8 (146.2)	−2.9	<.001
Increase	612 (21)	216.8 (141.2)	222.1 (144.0)	2.4	<.01
No change	905 (30)	263.3 (168.9)	262 (167.6)	−0.5	.02
**Sodium density, mg/100 g**					
Decrease	1,462 (49)	629.2 (375.5)	521.8 (314.5)	−17.1	<.001
Increase	612 (21)	536.9 (327.3)	613.4 (364.9)	14.2	<.001
No change	905 (30)	647.1 (408.4)	647.1 (408.4)	0.0	.78
**Sales, annual equivalized**					
Decrease	1,462 (49)	4,841,548.7 (3,966,836)	4,510,680.7 (3,736,687)	−6.8	<.001
Increase	612 (21)	3,645,839.3 (2,845,116)	3,364,883.9 (2,506,009.5)	−7.7	<.001
No change	905 (30)	4,188,194.8 (3,425,053)	4,129,743.8 (3,465,828)	−1.4	.50

Mean (SD) annual equivalized sales units declined significantly by 5.4% (*P* < .001), from 4,397,420.0 (3,591,787) in 2009 to 4,159,564.4 (3,475,658) in 2015. Sales decreased among 60% of products (n = 1,801), increased among 37% of products (n = 1,106) and did not change among 2% of products (n = 72). Products that decreased or increased calorie density demonstrated significant declines in sales units from 2009 to 2015. For decreases, the decrease was 8.2% (*P* < .001) (Figure 1); mean (SD) decrease from 4,307,499.3 (3,609,349) units in 2009 to 3,955,056.7 (3,460,379) units in 2015. For increases, the decrease was 5.9% (*P *= .02); mean (SD) decrease from 4,478,441.1 (3,498,997) units in 2009 to 4,214,948.1 (3,098,732) units in 2015. Products that decreased or increased sodium density demonstrated significant declines in sales units from 2009 to 2015. For decreases, the decrease was 6.8% (*P* < .001) (Figure 2); mean (SD) decrease from 4,841,548.7 (3,966,836) units in 2009 to 4,510,680.7 (3,736,687) units in 2015. For increases, the decrease was 7.7% (*P* < .001); mean (SD) decrease from 3,645,839.3 (2,845,116) units in 2009 to 3,364,883.9 (2,506,009.5) in 2015.

The mean difference in sales was not significant (*P* = .53) among products that decreased versus increased in calorie density (−352,442.7 kcal/100 g for products that decreased vs −263,493.0 kcal/100 g for products that increased).The difference also was not significant among products that decreased versus increased in sodium density (*P* = .66) (−330,868 mg/100 g for products that decreased vs −280,955 mg/100 g for products that increased).

## Discussion

What people eat and how much they eat contribute to deaths from heart disease, stroke, and type 2 diabetes (1). Our study found that sodium, calories, and serving size all decreased significantly among top-selling products on the market from 2009 to 2015. Calls for reformulation have raised concerns about the risk of unintended consequences, particularly whether manufacturers would increase calories through the addition of sugar or fats to compensate for reductions in sodium and vice versa (6). On average, this tradeoff did not occur: a decrease in calorie density or sodium density did not correspond to an increase in the other. In fact, products with reductions in calorie density also demonstrated reductions in sodium density and vice versa. Instead of there being unintended negative nutritional consequences of companies’ reformulating for one nutrient, a net benefit was observed. That is good news for consumers.

Among dietary factors, high sodium is estimated to be the greatest potential contributor to deaths because of the role it plays in raising blood pressure and the risk that blood pressure poses to heart health (1). Sodium density decreased significantly by 6%. If translated into individual behavior, a consumer who ate the same amount of processed food would have consumed on average 6% less sodium in 2015 than in 2009. These findings are consistent with results from analysis of changes in a larger, nonmatched sample of processed foods in 2009 and 2015 (5). It may reflect achievements by major processed food companies to lower sodium content in products as part of NSRI. The observed reductions across many metacategories are evidence that industry operating in different sectors can lower the sodium and calorie content of products over a 5-year period.

In contrast to sodium, calories per serving and calorie density decreased only modestly (−2% and −1.1%, respectively). Declines in calorie density were smaller and less widespread than the changes we observed in sodium density and, although significant, may reflect normal variation in product formulation over time. Greater declines were observed in products in which sodium reduction also occurred, which indicates that when processed food manufacturers reformulated products, they tackled key calorie contributors in addition to sodium.

The limited changes in calorie density are more modest than findings from an evaluation of the Healthy Weight Commitment Foundation, which found substantial reductions in calories in processed foods sold from 2007 to 2012 (7). As part of the Healthy Weight Commitment Foundation marketplace pledge, 16 major food and beverage companies committed to remove 1.5 trillion calories from the US marketplace from 2007 to 2015 (8). An independent evaluation found that by 2012 these companies had already exceeded their goal by more than 400% (7). These reductions could be attributed to a combination of industry-led strategies, such as introducing lower-calorie products, discontinuing higher-calorie products, changing package size, adjusting price, or reformulating products, and to independent consumer decisions possibly related to public health education. Our analysis is more recent, is focused on product reformulation in the same food products for NSRI categories only, and suggests that reformulation was not a key strategy used to lower calorie content among food. Continued monitoring will help the industry and the public health community to understand if progress across foods and beverages is sustained and which strategies are most enduring.

During the time period examined, changes in serving size were observed in many of the reformulated products. Serving size was reduced overall with greater declines in products that increased both calorie density and sodium density. The variation in serving size of similar products or the same product over time may make it harder for consumers to gauge other nutritional differences. Comparing labels for products with different serving sizes can require complicated mathematics for the average shopper; it would be ideal if similar products used the same serving size to make it easier for consumers to compare labels. Because of the variance of serving size across similar products and in amounts actually consumed, it is important for nutrition surveillance to assess changes in both the per-serving and density metrics to capture the full picture of reformulation.

Sales of products decreased overall from 2009 to 2015 (−5.4%), but the decrease in sales was not disproportionately concentrated among products that decreased calories or sodium. This is consistent with findings that lower calorie products can drive sales growth for food companies (9). It is also in line with recent consumer research that shows that shoppers are comparing labels to choose low-sodium products and are cutting back on high-sodium food (10, 11). These findings are relevant in light of the release of draft voluntary sodium targets from the Food and Drug Administration (FDA) for a wide range of foods and the ability of the food industry to follow them while continuing to maintain product sales (6). The FDA’s proposed 2-year sodium reduction targets are based on sodium levels in 2010, which is one year after the baseline data was collected for our analysis, suggesting that the findings from this analysis have bearing on the ease with which industry could meet the targets.

Ours is one of the first studies to explore changes in key components of US processed foods related to health and is a unique analysis of concurrent changes in serving size, calorie density, sodium density, and sales. Tracking changes among the same products that were chosen systematically based on high sales at 2 points in time provides unique insight into the complex changes in the food supply. However, our study had limitations. Nutrition data were not available for every top-selling product on the market in both 2009 and 2015 or for private-label products. A recent study found that sodium, total fat, and total sugar concentrations do not differ significantly between private-label and national food brands (12). Products sold through foodservice channels, Walmart, warehouse-style retailers, military commissaries, and small stores with less than $2 million annual sales revenue were not included. Walmart has reported reducing sodium and added sugar in national brands and in its GreatValue products as it worked towards providing healthier foods from 2011 to 2015 (13). The absence of data on private-label products and products sold by Walmart decreases the generalizability of our study findings. Our sample included food categories that contribute to sodium intake. Although dietary sodium intake comes from a wide range of sources, not all food categories were included; thus, reformulation in some foods (eg, beverages, yogurt, some desserts) was not captured. Finally, food companies could be reducing calorie density by replacing healthier fats with carbohydrates; an analysis of macronutrient composition of foods would be a valuable next step for research.

To optimize health, the ideal diet should be made up primarily of whole foods, such as fruits, vegetables, and legumes. The reality is that many Americans eat substantial amounts of processed foods, and the United States has a large food industry built to meet and foster consumer demand. Improving the healthfulness of processed food is important given Americans’ reliance on it. Food producers use a combination of economical ingredients, including sodium, sugar, and fat, to make food appealing, shelf-stable, and inexpensive (14). Consuming fewer calories and less sodium is challenging and requires a shift in food choices and eating patterns. As the public health community and consumers continue to push for products that allow consumers the choice to eat healthfully, it remains critical to monitor changes in the food supply to ensure that new formulations are truly healthier. The results of our study show that reductions in calorie density and sodium density occurred in tandem, suggesting that manufacturers can reformulate for more than one health goal at the same time. This finding helps mitigate the concern that the benefits of a reduction in one area would be offset by an increase in the other, although this may vary by food type. Our findings show that food companies can reformulate products to be healthier within a 5-year period without a negative effect on sales. Continued reductions are necessary to achieve population-wide reductions in sodium intake and consumption of excess calories, requiring active engagement of the food industry. The pace and scope of reduction must accelerate to improve population health.

## References

[1] Micha R , Peñalvo JL , Cudhea F , Imamura F , Rehm CD , Mozaffarian D . Association between dietary factors and mortality from heart disease, stroke, and type 2 diabetes in the United States. JAMA 2017;317(9):912–24. 10.1001/jama.2017.0947 28267855 PMC5852674

[2] Huth PJ , Fulgoni VL , Keast DR , Park K , Auestad N . Major food sources of calories, added sugars, and saturated fat and their contribution to essential nutrient intakes in the U.S. diet: data from the National Health and Nutrition Examination Survey (2003–2006). Nutr J 2013;12(116):116. 10.1186/1475-2891-12-116 23927718 PMC3751311

[3] Drewnowski A , Rehm CD . Energy intakes of US children and adults by food purchase location and by specific food source. Nutr J 2013;12(59):59. 10.1186/1475-2891-12-59 23656639 PMC3658962

[4] Drewnowski A , Rehm CD . Sodium intakes of US children and adults from foods and beverages by location of origin and by specific food source. Nutrients 2013;5(6):1840–55. 10.3390/nu5061840 23760055 PMC3725480

[5] Curtis CJ , Clapp J , Niederman SA , Ng SW , Angell SY . US food industry progress during the National Salt Reduction Initiative: 2009–2014. Am J Public Health 2016;106(10):1815–9. 10.2105/AJPH.2016.303397 27552265 PMC5024394

[6] US Food and Drug Administration. Draft guidance for industry: voluntary sodium reduction goals: target mean and upper bound concentrations for sodium in commercially processed, packaged, and prepared foods. http://www.fda.gov/Food/GuidanceRegulation/GuidanceDocumentsRegulatoryInformation/ucm494732.htm. Accessed May 18, 2017.

[7] Ng SW , Slining MM , Popkin BM . The Healthy Weight Commitment Foundation pledge: calories sold from U.S. consumer package goods, 2007–2012. Am J Prev Med 2015;47:508–19. 10.1016/j.amepre.2014.05.029 PMC4171694 25240967

[8] Food and beverage companies surpass 2015 goal of reducing calories in the U.S. three years ahead of schedule. Healthy Weight Commitment Foundation. May 30, 2013. http://www.healthyweightcommit.org/wp-content/uploads/2016/03/05302013_HWCF_PR.pdf. Accessed May 18, 2017.

[9] Cardello H , Wolfson J . Lower-calorie foods and beverages drive Healthy Weight Commitment Foundation companies’ sales growth: interim report. Hudson Institute. http://www.hudson.org/content/researchattachments/attachment/1107/lowercalhealthyweightcommitment–may2013.pdf?wb48617274=AC279019. Accessed May 18, 2017.

[10] International Food Information Council Foundation. What’s your health worth? Food and Health Survey 2015. http://www.foodinsight.org/sites/default/files/2015-Food-and-Health-Survey-Full-Report.pdf. Accessed May 18, 2017.

[11] Food Marketing Institute. Prevention Magazine and Food Marketing Institute reveal 20th annual “Shopping for Health” survey results. http://www.fmi.org/news-room/latest-news/view/2012/07/17/prevention-magazine-and-food-marketing-institute-reveal-20th-annual-shopping-for-health-survey-results. 2012. Accessed May 18, 2017.

[12] Ahuja JKC , Pehrsson PR , Cogswell M . A comparison of concentrations of sodium and related nutrients (potassium, total dietary fiber, total and saturated fat, and total sugar) in private-label and national brands of popular, sodium-contributing, commercially packaged foods in the United States. J Acad Nutr Diet 2017;117(5):770–777.e17. 10.1016/j.jand.2016.12.001 28169210

[13] Walmart. Our commitments. http://corporate.walmart.com/global-responsibility/hunger-nutrition/our-commitments. Accessed May 18, 2017.

[14] Moss M . Salt sugar fat: How the food giants hooked us. New York (NY): Random House; 2013.

